# Platelet-Rich Plasma in the Management of Temporomandibular Joint Pain in Young Adults With Temporomandibular Disorder

**DOI:** 10.7759/cureus.55609

**Published:** 2024-03-05

**Authors:** Santosh Kumar Mathpati, Gourav Jain, Vijay Mishra, Atul K Singh, Rahul Mishra, Bipin K Yadav

**Affiliations:** 1 Department of Oral and Maxillofacial Surgery, Navodaya Dental College and Hospital, Raichur, IND; 2 Department of Oral and Maxillofacial Surgery, Faculty of Dentistry, Uttar Pradesh University of Medical Sciences, Etawah, IND; 3 Department of Pedodontics and Preventive Dentistry, Faculty of Dentistry, Uttar Pradesh University of Medical Sciences, Etawah, IND; 4 Department of Periodontology, Faculty of Dentistry, Uttar Pradesh University of Medical Sciences, Etawah, IND

**Keywords:** pain, dentistry, regenerative medicine, platelet-rich plasma, tmj pain, temporomandibular disorder

## Abstract

Background: Temporomandibular disorder (TMD) encompasses a range of conditions affecting the temporomandibular joint (TMJ) and associated structures, with TMJ pain being a prevalent symptom. Conventional management strategies have limitations, which require the exploration of innovative interventions. Platelet-rich plasma (PRP), known for its regenerative properties, presents a potential therapeutic avenue. This study aims to investigate the effectiveness of PRP in reducing the pain associated with mild TMJ in young adults.

Methodology: Participants (*n* = 128) aged 18 to 35 years with mild TMD were evenly randomized into PRP treatment and placebo control groups. PRP was prepared using a standardized protocol, and intra-articular injections were administered. Placebo injections mimic PRP. Follow-up evaluations were carried out at four and eight weeks after the intervention.

Results: The study successfully randomized comparable groups, and the PRP treatment group experienced a significant reduction in TMJ pain (visual analog scale [VAS] score: 6.8 ± 1.2 to 2.1 ± 1.0 at eight weeks, *P *< 0.001). The PRP treatment also increased the largest opening of the mouth (from 38.2 ± 2.5 to 43.5 ± 3.1, *P* < 0.001) and the number of lateral movements (12.3 ± 1.5 to 14.9 ± 2.0, *P* < 0.001), while the placebo group had very few changes. Positive patient-reported outcomes on daily activities were observed, with no serious complications reported in either group.

Conclusions: This study provides evidence supporting the efficacy of PRP in reducing TMJ pain, improving jaw function, and improving quality of life in young adults with mild TMD. The results underscore the potential of PRP as a minimally invasive intervention for TMJ disorders.

## Introduction

Temporomandibular disorder (TMD) is a group of conditions that affect the temporomandibular joint (TMJ), the muscles that help you chew, and the structures that are connected to them [1,2]. TMJ pain is one of its symptoms that is common and debilitating, frequently accompanied by functional restrictions and having a significant impact on the quality of life of those who are affected by it [3]. Although the precise etiology of TMD remains multifactorial, contributing factors include trauma, occlusal abnormalities, and psychosocial stressors [4,5]. Conventional treatment of TMJ pain ranges from conservative measures, such as physical therapy and analgesics, to more invasive interventions, such as intra-articular injections and surgical procedures [6,7]. Despite these options, a subset of people continue to experience persistent symptoms, emphasizing the need for innovative and effective therapeutic strategies.

Platelet-rich plasma (PRP), a biologic autologous product derived from whole blood, has gained attention for its regenerative and anti-inflammatory properties [8]. Rich in growth factors, cytokines, and other bioactive molecules, PRP is effective in promoting tissue healing and reducing inflammation in various musculoskeletal conditions [9]. Although studies have explored its application in various medical conditions [10], its potential role in alleviating TMJ pain in individuals with mild TMD remains relatively unexplored. This prospective, randomized, double-blind, placebo-controlled clinical trial aims to investigate the effectiveness of PRP in reducing TMJ pain associated with mild cases of TMD in young adults. The rationale for this study comes from the need for novel and minimally invasive interventions that address the underlying pathophysiology of TMJ pain. By rigorously examining the outcomes of PRP treatment, we aimed to contribute valuable information on its potential as an alternative or adjunctive therapeutic option for people with TMJ pain.

The significance of this study lies in its potential to advance the field's understanding of PRP's role in managing TMJ pain. If proven effective, PRP could offer a minimally invasive and autologous approach to alleviate TMJ pain and improve the overall well-being of individuals with mild TMD [10]. The results of this trial may pave the way for further investigations, refine treatment algorithms, and provide clinicians with additional tools to address the multifaceted nature of TMJ disorders.

## Materials and methods

The study's recruitment was designed to enroll a diverse cohort of young adults aged between 18 and 35 years. Potential participants were identified through dental clinics, university health centers, and community outreach programs. A detailed informed consent form was provided to each individual, explaining the nature of the study, potential risks, and benefits. The study adhered to the principles outlined in the Declaration of Helsinki, and the study protocol received approval from the Institutional Review Board (IRB) before the start of the study with IRB number IEC/UPUMS/2021/IR/23.

The inclusion criteria required participants to have a diagnosis of TMD determined through clinical evaluation and adherence to diagnostic criteria [11]. The presence of self-reported TMJ pain was a crucial inclusion criterion. Willingness to actively participate in the study, including attendance at scheduled follow-up evaluations. Individuals with a history of bleeding disorders or platelet dysfunction were excluded from participation due to potential complications related to blood collection and PRP preparation. Pregnant or lactating women were excluded to ensure the safety of participants and unborn or nursing children. Individuals who had undergone TMJ surgery or received joint injections within the last six months were excluded to minimize confounding variables. Participants with systemic inflammatory disorders, which could affect TMJ pain, as well as those with stress factors potentially skewing study results, were also excluded.

The screening process involved a comprehensive review of the patient's medical history, clinical examinations performed by qualified dental professionals, and diagnostic imaging when necessary. Potential participants who met the inclusion criteria underwent detailed discussions with the research team to ensure their understanding of the study requirements and commitment. Before the intervention, baseline evaluations were conducted to gather complete information on each participant's demographic details, medical history, TMJ pain characteristics, and baseline pain levels using the visual analog scale (VAS) [12]. These baseline assessments served as a foundation for comparison throughout the study.

When the inclusion criteria were met, the 128 participants were assigned to either the PRP treatment group or the placebo control group using a computer-generated sequence. A double-blind design was implemented to minimize bias. Neither the participants nor the clinicians administering the interventions were aware of the assignment of the group. Placebo injections were meticulously designed to be indistinguishable from PRP injections, further ensuring the effectiveness of blinding.

PRP was meticulously prepared following a standardized protocol. Participants in this group underwent a blood collection process, typically in the antecubital vein, using aseptic techniques. The collected blood was then subjected to centrifugation, allowing the separation of the platelet-rich component. The resulting PRP was carefully extracted and prepared for intraarticular injection. The prepared PRP was administered to the affected TMJ by a trained clinician specializing in the procedure. When necessary, imaging modalities and anatomical landmarks guided the injection. The goal was to ensure precise delivery of PRP to the target site within the TMJ. Participants in the PRP treatment group were scheduled for follow-up evaluations at four weeks and eight weeks after intervention. These evaluations included clinical examinations, imaging studies if indicated, and the collection of patient-reported results. The assessments aimed to evaluate the sustained effects of the PRP intervention on the reduction of TMJ pain, jaw function, and any adverse events.

Participants in the control group received intraarticular injections of a placebo solution, typically consisting of normal saline. The placebo solution was intentionally crafted to mimic the appearance of the PRP, ensuring that both the participant and the administering clinician remained blinded to the intervention. Similarly to the PRP treatment group, participants in the placebo control group underwent follow-up evaluations at four weeks and eight weeks post-intervention. These evaluations mirrored those performed for the PRP group and included clinical evaluations, imaging studies if necessary, and the collection of patient-reported outcomes. The purpose was to compare the results between the two groups, discerning any differences in the reduction of TMJ pain and related parameters.

Outcome measures

Primary Outcome: TMJ Pain Reduction

The primary objective of this study was to evaluate the efficacy of PRP in reducing TMJ pain. The reduction in TMJ pain was meticulously assessed using VAS scores. Participants' TMJ pain levels were measured at two distinct time points: four weeks and eight weeks after intervention. These time intervals were strategically chosen to capture both the short-term and sustained effects of PRP treatment. The VAS, a validated and widely used pain assessment tool, was used. Participants were asked to indicate their perceived level of TMJ pain by marking a point along a continuous line, ranging from *no pain* to *worst imaginable pain*. Pain scores were then quantified for statistical analysis.

Secondary Results

Changes in jaw function were assessed using objective measurements, including maximal mouth opening and lateral excursions. Baseline measurements were established during the initial assessment. The maximum distance between the upper and lower incisors was measured using a calibrated device. The range of lateral movements during jaw opening was recorded, providing information on possible improvements or limitations in jaw function. These measurements were repeated at four- and eight-week follow-up assessments to track changes in jaw function over time.

Patient-reported outcomes were collected to gain insight into the impact of TMJ pain on daily activities. Participants were asked to complete standardized questionnaires addressing pain interference with various daily activities like eating and chewing, speaking, sleeping, and the overall quality of life. The responses were quantitatively analyzed to identify patterns and trends, providing a comprehensive understanding of how TMJ pain influenced the daily lives of participants.

Throughout the study, a meticulous monitoring protocol was in place to document adverse events or complications associated with the interventions related to PRP treatment or placebo injections. Participants were encouraged to report any unexpected symptoms or problems. Additionally, scheduled follow-up assessments included specific inquiries about potential adverse events to ensure comprehensive documentation. Adverse events were systematically classified according to severity, ranging from mild discomfort to serious complications. This classification system facilitated a nuanced analysis of safety outcomes associated with interventions.

Statistical analysis

Statistical analysis was performed using IBM SPSS Statistics for Windows, Version 24.0 (IBM Corp., Armonk, NY). Continuous variables were analyzed using t-tests or nonparametric equivalents, and categorical variables were assessed using chi-square tests. A significance level of *P* < 0.05 was established.

## Results

Table 1 reveals that randomization successfully created comparable groups.

**Table 1:** Demographic characteristics of participants. PRP, platelet-rich plasma; SD, standard deviation

Characteristics	PRP treatment group	Placebo control group
Total participants	64	64
Age (mean ± SD)	28.5 ± 3.2	29.1 ± 3.5
Gender (Male/Female)	30/34	32/32

The mean age was 28.5 ± 3.2 years in the PRP treatment group and 29.1 ± 3.5 years in the placebo control group. The gender distribution was also balanced, with 30 men and 34 women in the PRP group and 32 men and 32 women in the placebo group.

The primary outcome demonstrates a statistically significant reduction in TMJ pain in the PRP treatment group. At four weeks, the mean VAS score decreased from 6.8 ± 1.2 (baseline) to 3.2 ± 1.5 (*P* < 0.05). At eight weeks, it decreased further to 2.1 ± 1.0 (*P* < 0.001). On the contrary, the placebo group showed no significant changes, with scores of 6.5 ± 1.1 at baseline, 5.9 ± 1.2 at four weeks, and 5.7 ± 1.3 at eight weeks (Table 2).

**Table 2:** Visual analog scale (VAS) scores for TMJ pain reduction. PRP, platelet-rich plasma; TMJ, temporomandibular joint

Time point	PRP treatment group (mean ± SD)	Placebo control group (mean ± SD)
Baseline	6.8 ± 1.2	6.5 ± 1.1
Four weeks	3.2 ± 1.5	5.9 ± 1.2
Eight weeks	2.1 ± 1.0	5.7 ± 1.3

Objective measurements revealed notable improvements in jaw function in the PRP treatment group. The maximum mouth opening increased from 38.2 ± 2.5 (baseline) to 43.5 ± 3.1 at 8 weeks (*P* < 0.001). Lateral excursions increased from 12.3 ± 1.5 (baseline) to 14.9 ± 2.0 at eight weeks (*P* < 0.001). On the contrary, the placebo group showed minimal changes, with no significant differences at any time (Table 3).

**Table 3:** Changes in jaw function parameters. PRP, platelet-rich plasma

Parameter	PRP treatment group (mean ± SD)	Placebo control group (mean ± SD)
Maximal mouth opening	Baseline: 38.2 ± 2.5	Baseline: 38.0 ± 2.2
	Four weeks: 42.1 ± 2.8	Four weeks: 37.9 ± 2.1
	Eight weeks: 43.5 ± 3.1	Eight weeks: 38.2 ± 2.5
Lateral excursions	Baseline: 12.3 ± 1.5	Baseline: 12.5 ± 1.3
	Four weeks: 14.2 ± 1.8	Four weeks: 12.6 ± 1.2
	Eight weeks: 14.9 ± 2.0	Eight weeks: 12.7 ± 1.5

Participants in the PRP treatment group reported significant improvements in various daily activities. For example, in eating and chewing, the scores decreased from 6.2 ± 1.4 (baseline) to 2.2 ± 1.2 at eight weeks (*P* < 0.001). Similar improvements in speaking, sleeping, and overall quality of life were observed. On the contrary, the placebo group showed marginal changes, with no significant differences between the assessed activities (Table 4).

**Table 4:** Patient-reported outcomes. PRP, platelet-rich plasma

Activity	PRP treatment group (mean ± SD)	Placebo control group (mean ± SD)
Eating and chewing	Baseline: 6.2 ± 1.4	Baseline: 6.0 ± 1.3
	Four weeks: 3.1 ± 1.6	Four weeks: 5.8 ± 1.2
	Eight weeks: 2.2 ± 1.2	Eight weeks: 5.6 ± 1.4
Speaking	Baseline: 5.8 ± 1.3	Baseline: 5.9 ± 1.2
	Four weeks: 3.4 ± 1.5	Four weeks: 5.7 ± 1.1
	Eight weeks: 2.6 ± 1.0	Eight weeks: 5.5 ± 1.3
Sleeping	Baseline: 6.0 ± 1.2	Baseline: 5.8 ± 1.1
	Four weeks: 3.0 ± 1.4	Four weeks: 5.9 ± 1.2
	Eight weeks: 1.8 ± 0.9	Eight weeks: 6.0 ± 1.1
Overall quality of life	Baseline: 5.9 ± 1.1	Baseline: 5.7 ± 1.0
	Four weeks: 2.8 ± 1.2	Four weeks: 5.6 ± 1.3
	Eight weeks: 1.5 ± 0.8	Eight weeks: 5.5 ± 1.2

The incidence of adverse events in the PRP treatment group was minimal. Mild discomfort was reported by 4 (6.3%), localized swelling by 2 (3.1%), and headache by 1 (1.6%). Importantly, 57 (89.1%) reported no adverse events. In the placebo group, 3 (4.7%) reported mild discomfort, 1 (1.6%) reported localized swelling, 2 (3.1%) reported headache, and 58 (90.6%) reported no adverse events. No serious complications were reported in either group (Table 5).

**Table 5:** Adverse events and complications. PRP, platelet-rich plasma

Event category	PRP treatment group, *n* (%)	Placebo control group, *n* (%)
Mild discomfort	4 (6.3%)	3 (4.7%)
Localized swelling	2 (3.1%)	1 (1.6%)
Headache	1 (1.6%)	2 (3.1%)
No adverse events	57 (89.1%)	58 (90.6%)

## Discussion

This study investigated the efficacy of PRP in reducing TMJ pain among young adults with mild TMD. The results suggest that PRP treatment, compared to placebo, led to significant improvements in TMJ pain, jaw function, and patient-reported outcomes. According to the VAS scores, there was a significant decrease in TMJ pain in the PRP treatment group. At four weeks after the intervention, the mean VAS score decreased from 6.8 ± 1.2 (the baseline) to 3.2 ± 1.5, and at eight weeks, it decreased to 2.1 ± 1.0. These findings are consistent with previous studies that have demonstrated the potential analgesic effects of PRP in various musculoskeletal conditions [13-15]. The proposed mechanism involves the release of platelet growth factors, modulating inflammation, and promoting tissue repair [16]. In the context of TMJ pain, the results suggest that PRP may serve as an effective intervention for symptom management.

The improvements in the parameters of jaw function further support the potential therapeutic benefits of PRP. Maximal mouth opening and lateral excursions increased significantly compared to baseline after PRP treatment. These objective measurements align with the observed reduction in TMJ pain and suggest a multifaceted positive impact on jaw mobility. This is consistent with the study by Sousa et al., where patients who received PRP showed an improvement in maximum pain-free mouth opening after treatment. In particular, the best results were observed in the PRP arm after 6 months [17]. PRP injections may reduce joint pain and sound and improve joint range of motion because PRP injections have anti-inflammatory and analgesic properties. PRP restores intraarticular hyaluronic acid levels, increases chondrocyte glycosaminoglycan synthesis, and balances joint angiogenesis. However, a standardized protocol for the preparation and application of PRP must be established [18]. These observations also align with studies investigating PRP in orthopedic applications, where it has shown promise in promoting tissue regeneration and reducing functional limitations [19, 20].

Patient-reported results, which focused on activities such as eating, speaking, sleeping, and overall quality of life, showed substantial improvements in the PRP treatment group. Participants reported a decrease in pain interference with daily activities, reflecting a comprehensive improvement in their functional well-being. Similar improvements have been reported in studies investigating PRP for conditions such as osteoarthritis [21]. The safety profile of PRP in this study appears favorable, with a low incidence of mild adverse events such as discomfort, localized swelling, and headache. These events were transient and consistent with previous literature suggesting that PRP is generally well tolerated [22,23]. Importantly, no serious complications that required intervention were reported, highlighting the safety of PRP in the context of TMJ interventions.

Despite promising findings, this study has several limitations. First, the duration of the study may not capture the long-term effects and potential complications associated with PRP treatment. Additionally, the blinding process, while meticulously designed, may not completely eliminate bias, and future studies could explore alternative blinding strategies. Additionally, inclusion criteria focused on young adults with mild TMD, limiting the generalizability of the findings to other age groups and the severity of TMD. Based on the current study, future research should focus on optimizing PRP preparation protocols, considering variations in platelet concentration and the inclusion of adjunctive substances. Comparative effectiveness studies pitting PRP against other therapeutic modalities could provide a more comprehensive understanding of its place in the treatment of TMJ disorders.

Exploring the molecular and cellular mechanisms underlying the observed improvements in TMJ pain and function would deepen our understanding of the therapeutic actions of PRP. Additionally, investigating long-term outcomes and potential predictors of response to treatment could guide personalized treatment approaches. While the study provides promising findings regarding the effectiveness of PRP treatment for TMJ pain, the identified limitations underscore the need for further research to address these constraints and enhance the validity and generalizability of the findings.

## Conclusions

In conclusion, this prospective, randomized, double-blind, placebo-controlled clinical trial shows that PRP may help young adults with mild TMD feel less pain and have better outcomes related to their condition. The findings align with the regenerative properties attributed to PRP in musculoskeletal conditions. Overall, these findings suggest that PRP therapy holds promise as an effective and safe intervention for managing TMJ pain in young adults with mild TMD. While acknowledging the study's limitations, these results contribute to the evolving landscape of the management of TMJ disorders. More research is needed, particularly on long-term outcomes and refinement of treatment protocols, to solidify the role of PRP in therapeutics for TMJ. 
